# Supplementary material for: Patterns of annual and seasonal immune investment in a temporal reproductive opportunist
Source: Proc Biol Sci. 2020 Jun 24;287(1929):20192993. doi: 10.1098/rspb.2019.2993 (PMC7329054; doi:10.1098/rspb.2019.2993)
Supplement: Supplemental Figures and Tables [file rspb20192993supp1.pdf]

Supplemental Figures and Tables

Schultz et al. 2020

Table S1: Number of samples per time period (cone year and season) for each response. Not all responses were measured for all birds; no birds were recaptured. Cone year columns (2010-2013) include only samples from summer. Season columns (Summer-Spring) include only samples from Cone Year 2011. Responses: Lysis (positive hemolysis score: F=0, T=1), Agglut (agglutination score), PIT54 (mg/mL), WBC (Prop Leukocytes/Erythrocytes), Lymp (Prop Lymphocytes/Leukocytes), Mono (Prop Monocytes/Leukocytes).

Response	2010	2011	2012	2013	Summer	Autumn	Winter	Spring
Lysis	29	55	96	13	55	11	98	26
Agglut	29	55	96	13	55	11	98	26
PIT54	0	54	92	13	54	10	90	25
WBC	29	52	84	12	52	10	79	28
Lymp	29	53	84	12	53	10	77	27
Mono	29	53	84	12	53	10	77	27

Table S2: Summary of samples per time interval for each vocal type. The majority of samples were of vocal type 5 (72%), followed by type 2 (10.3%), type 4 (8.8%), unknown (NA, 4.7%), and type 3 (4.1%).

Cone Year	Season	Type 2	Type 3	Type 4	Type 5	Type NA
2010	Summer				30	
2011	Summer	2	1	12	39	2
2011	Autumn				11	
2011	Winter	4	2	11	74	8
2011	Spring	2	7		19	
2012	Summer	23	4	6	63	5
2013	Summer	4		1	8	1

Table S3: List of considered covariates. Several covariates were excluded from consideration due to technical limitations: missing or sparse observations (hematocrit and vocal type), and strong association with season (site name and time of capture). Tmax was also excluded due to a strong positive correlation with Tmin.

Name	Units.and.Values	Notes
Cone.year	Integer: 2010-2013	Cone growing season (start of year): June 1 through subsequent May 30
Season	Category: Su, F, W, Sp	Astronomical season
Precip	mm	Total daily precipitation at Moose, WY
Tmin, Tmax	degrees C	Minimum and maximum daily temperature at Moose, WY
Tdiff	degrees C	Tmax - Tmin
Sex	Category: M, F	Determined from plumage
Age	Category: HY, AHY	Determined from plumage, skull: hatch year (HY) or after hatch year (AHY)
Body.molt	Integer: 0-3	Score: contour feather moult intensity
CP/BP	Z-score (within sex)	Reproductive condition: cloacal protuberance length (M) or brood patch score (F)
Fat	Integer: 0-5	Score: furcular and abdominal subcutaneous fat
Ff	Integer: 0-7	Number of actively growing flight feathers (out of 18 total)
R.mass	grams	Body condition: tarsus mass regression residuals
Capture.dur	log10 minutes	Capture duration: length of time from capture to release

Table S4: **Cone year:** Expected marginal means for each model that includes time period as a covariate (horizontal lines separate models). Group: Honest Significant Difference groupings (within model, α=0.05), using Tukey adjustment. All estimates are shown in the units of the response. See also Fig. 2.

Response	Cone.year	Est	SE	95% CI	Group	nObs
Lysis	2010	0.331	0.090	0.15-0.58	a	28
Lysis	2011	0.684	0.064	0.51-0.82	b	54
Lysis	2012	0.165	0.039	0.09-0.28	a	96
Lysis	2013	1.000	0.000	0.00-1.00	ab	13
Agglut	2010	10.404	0.434	9.31-11.50	a	27
Agglut	2011	7.275	0.335	6.43-8.12	b	55
Agglut	2012	9.755	0.237	9.16-10.35	a	96
Agglut	2013	10.444	0.593	8.95-11.94	a	13
PIT54	2011	0.212	0.043	0.11-0.32	a	53
PIT54	2012	0.405	0.031	0.33-0.48	b	91
PIT54	2013	0.293	0.080	0.10-0.49	ab	13
Lymp	2010	0.218	0.042	0.11-0.32	a	28
Lymp	2011	0.425	0.032	0.34-0.50	b	52
Lymp	2012	0.524	0.024	0.46-0.58	b	84
Lymp	2013	0.577	0.065	0.41-0.74	b	12
Mono	2010	0.432	0.046	0.31-0.55	a	28
Mono	2011	0.217	0.044	0.11-0.33	b	53
Mono	2012	0.150	0.043	0.04-0.26	b	84
Mono	2013	0.118	0.059	-0.03-0.27	b	12
WBC	2010	0.004	0.000	0.00-0.00	ab	28
WBC	2011	0.005	0.000	0.00-0.01	a	51
WBC	2012	0.003	0.000	0.00-0.00	b	84
WBC	2013	0.005	0.001	0.00-0.01	ab	12

Table S5: **Season:** Expected marginal means for each model that includes time period as a covariate (horizontal lines separate models). Group: Honest Significant Difference groupings (within model, α=0.05), using Tukey adjustment. All estimates are shown in the units of the response. See also Fig. 2.

Response	Season	Est	SE	95% CI	Group	nObs
Lysis	Autumn	0.769	0.131	0.35-0.95	a	11
Lysis	Spring	0.364	0.139	0.11-0.72	a	18
Lysis	Summer	0.762	0.206	0.16-0.98	a	55
Lysis	Winter	0.285	0.143	0.07-0.70	a	96
Agglut	Autumn	8.372	0.743	6.50-10.24	a	11
Agglut	Spring	8.685	0.621	7.12-10.25	a	18
Agglut	Summer	8.251	1.172	5.30-11.20	a	55
Agglut	Winter	8.508	0.722	6.69-10.32	a	98
PIT54	Autumn	0.185	0.055	0.05-0.32	a	10
PIT54	Spring	0.175	0.045	0.06-0.29	a	17
PIT54	Summer	0.238	0.060	0.09-0.39	a	53
PIT54	Winter	0.234	0.041	0.13-0.34	a	90
Lymp	Autumn	0.180	0.164	-0.23-0.59	a	10
Lymp	Spring	0.756	0.065	0.59-0.92	b	19
Lymp	Summer	0.627	0.147	0.26-1.00	ab	52
Lymp	Winter	0.491	0.098	0.24-0.74	ab	74
Mono	Autumn	0.442	0.101	0.19-0.70	a	10
Mono	Spring	0.017	0.042	-0.09-0.12	b	19
Mono	Summer	0.135	0.080	-0.07-0.34	ab	53
Mono	Winter	0.142	0.060	-0.01-0.29	b	75
WBC	Autumn	0.005	0.001	0.00-0.01	a	10
WBC	Spring	0.002	0.001	0.00-0.00	a	19
WBC	Summer	0.002	0.001	-0.00-0.00	a	52
WBC	Winter	0.004	0.001	0.00-0.01	a	77

Table S6: LM regression coefficients for continuous (non-categorical) covariates (i.e., slopes). Horizontal lines denote separate models; within each model, estimates are ordered by p-value. For clarity, only models with $R^2 > 0.1$ are shown here, and estimates with p-value < 0.1 are bolded. Lysis: logistic GLM estimates back-transformed to linear scale, showing proportional change.

Response	Time	Covariate	Est	SE	P.value
**Lysis**	**Year**	**Ff**	**1.3**	**0.138**	**0.054**
Lysis	Year	Capture.dur	2.03	0.679	0.296
Lysis	Season	R.mass	1.12	0.0759	0.129
Lysis	Season	Precip	1.15	0.125	0.263
Lysis	Season	Fat	1.12	0.181	0.523
Lysis	Season	Ff	1.17	0.249	0.525
Lysis	Season	Tdiff	1.04	0.0788	0.579
Lysis	Season	Tmin	0.966	0.0711	0.631
Agglut	Year	Tdiff	0.0281	0.0652	0.667
Agglut	Year	Tmin	0.0195	0.0641	0.762
Agglut	Year	Body.molt	0.0097	0.196	0.961
**PIT54**	**Season**	**Precip**	**0.0287**	**0.0074**	**1.56e-04**
**PIT54**	**Season**	**R.mass**	**0.0127**	**0.0058**	**0.030**
**PIT54**	**Season**	**Capture.dur**	**-0.0659**	**0.0364**	**0.072**
PIT54	Season	Tmin	-0.0011	0.0042	0.793
PIT54	Season	Fat	0.00132	0.0138	0.924
Lymp	Year	Tdiff	-0.0107	0.00708	0.134
Lymp	Year	Capture.dur	-0.0795	0.0552	0.152
Lymp	Year	Precip	-0.00598	0.00584	0.307
Lymp	Year	Tmin	-0.00359	0.00653	0.584
**Lymp**	**Season**	**Capture.dur**	**-0.137**	**0.0649**	**0.036**
Lymp	Season	Ff	-0.049	0.0327	0.136
Lymp	Season	Tdiff	-0.0129	0.00994	0.197
Lymp	Season	Precip	-0.0212	0.0178	0.235
Lymp	Season	Tmin	-0.00927	0.00913	0.311
Lymp	Season	Cp.bp	0.0278	0.03	0.355
Lymp	Season	Body.molt	0.042	0.0517	0.419
Mono	Year	Tmin	-0.00487	0.00376	0.197
Mono	Year	Fat	0.00776	0.00898	0.389
Mono	Year	R.mass	-0.00193	0.00584	0.742
Mono	Season	Ff	0.0337	0.0208	0.108
Mono	Season	R.mass	-0.00532	0.00589	0.368
Mono	Season	Tmin	0.00182	0.00536	0.735
Mono	Season	Tdiff	-0.0012	0.00451	0.791
Mono	Season	Body.molt	-0.00231	0.0308	0.940
**WBC**	**Year**	**Capture.dur**	**-0.00137**	**0.000683**	**0.047**
WBC	Year	Tmin	0.000138	8.47e-05	0.106
WBC	Year	Tdiff	0.000137	8.48e-05	0.107
WBC	Year	Ff	0.000273	0.000179	0.128
WBC	Year	R.mass	-6.23e-05	7.91e-05	0.432
WBC	Year	Precip	3.92e-05	6.9e-05	0.571
**WBC**	**Season**	**Tdiff**	**0.000244**	**7.9e-05**	**0.002**
**WBC**	**Season**	**Precip**	**0.000386**	**0.000132**	**0.004**
**WBC**	**Season**	**Tmin**	**0.000181**	**7.1e-05**	**0.012**
WBC	Season	Ff	0.000452	0.000273	0.100
WBC	Season	Body.molt	-0.000147	0.000386	0.705

Table S7: Lysis: Type II Anova. Horizontal line shows separate models.

Time	Covariate	Df	LR Chisq	Pr(>Chisq)
Year	Cone.year	3	61.672	2.58e-13
Year	Ff	1	3.635	0.0566
Year	Capture.dur	1	1.141	0.286
Season	Season	3	6.456	0.0914
Season	R.mass	1	2.378	0.123
Season	Precip	1	1.286	0.257
Season	Ff	1	0.435	0.51
Season	Fat	1	0.410	0.522
Season	Tdiff	1	0.310	0.578
Season	Tmin	1	0.230	0.631

Table S8: Agglut: Type II Anova. Horizontal line shows separate models.

Time	Covariate	Df	F value	Pr(>F)
Year	Cone.year	3	18.678	1.3e-10
Year	Tdiff	1	0.186	0.667
Year	Tmin	1	0.092	0.762
Year	Body.molt	1	0.002	0.961
Year	Age	1	0.002	0.965
Year	Residuals	183		
Season	Tdiff	1	1.843	0.176
Season	Tmin	1	0.987	0.322
Season	Fat	1	0.143	0.705
Season	Season	3	0.092	0.964
Season	Residuals	175		

Table S9: Lymp: Type II Anova. Horizontal line shows separate models.

Time	Covariate	Df	F value	Pr(>F)
Year	Cone.year	3	14.403	2.15e-08
Year	Tdiff	1	2.273	0.134
Year	Capture.dur	1	2.069	0.152
Year	Precip	1	1.052	0.307
Year	Tmin	1	0.302	0.584
Year	Residuals	168		
Season	Season	3	4.574	0.00434
Season	Capture.dur	1	4.472	0.0362
Season	Ff	1	2.247	0.136
Season	Age	1	1.800	0.182
Season	Sex	2	1.683	0.19
Season	Tdiff	1	1.678	0.197
Season	Precip	1	1.424	0.235
Season	Tmin	1	1.032	0.311
Season	Cp.bp	1	0.861	0.355
Season	Body.molt	1	0.658	0.419
Season	Residuals	141		

Table S10: Mono: Type II Anova. Horizontal line shows separate models.

Time	Covariate	Df	F value	Pr(>F)
Year	Cone.year	3	19.921	4.36e-11
Year	Sex	2	2.100	0.126
Year	Tmin	1	1.678	0.197
Year	Age	1	1.392	0.24
Year	Fat	1	0.746	0.389
Year	R.mass	1	0.109	0.742
Year	Residuals	167		
Season	Season	3	5.492	0.00133
Season	Ff	1	2.620	0.108
Season	R.mass	1	0.815	0.368
Season	Sex	2	0.529	0.591
Season	Tmin	1	0.115	0.735
Season	Tdiff	1	0.070	0.791
Season	Body.molt	1	0.006	0.94
Season	Residuals	146		

Table S11: WBC: Type II Anova. Horizontal line shows separate models.

Time	Covariate	Df	F value	Pr(>F)
Year	Cone.year	3	7.238	0.000136
Year	Capture.dur	1	4.019	0.0466
Year	Tmin	1	2.638	0.106
Year	Tdiff	1	2.625	0.107
Year	Ff	1	2.342	0.128
Year	R.mass	1	0.621	0.432
Year	Precip	1	0.322	0.571
Year	Residuals	165		
Season	Tdiff	1	9.523	0.00242
Season	Precip	1	8.560	0.00397
Season	Tmin	1	6.492	0.0118
Season	Ff	1	2.734	0.1
Season	Season	3	1.949	0.124
Season	Body.molt	1	0.144	0.705
Season	Residuals	149		

Table S12: PIT54: Type II Anova. Horizontal line shows separate models.

Time	Covariate	Df	F value	Pr(>F)
Year	Cone.year	2	5.181	0.00668
Year	Capture.dur	1	5.766	0.0176
Year	Body.molt	1	1.793	0.183
Year	Tdiff	1	1.394	0.24
Year	Age	1	0.765	0.383
Year	Cp.bp	1	0.432	0.512
Year	Residuals	149		
Season	Precip	1	14.999	0.000156
Season	R.mass	1	4.802	0.0299
Season	Capture.dur	1	3.283	0.0719
Season	Season	3	0.861	0.463
Season	Tmin	1	0.069	0.793
Season	Fat	1	0.009	0.924
Season	Residuals	161		

Supplemental Figures

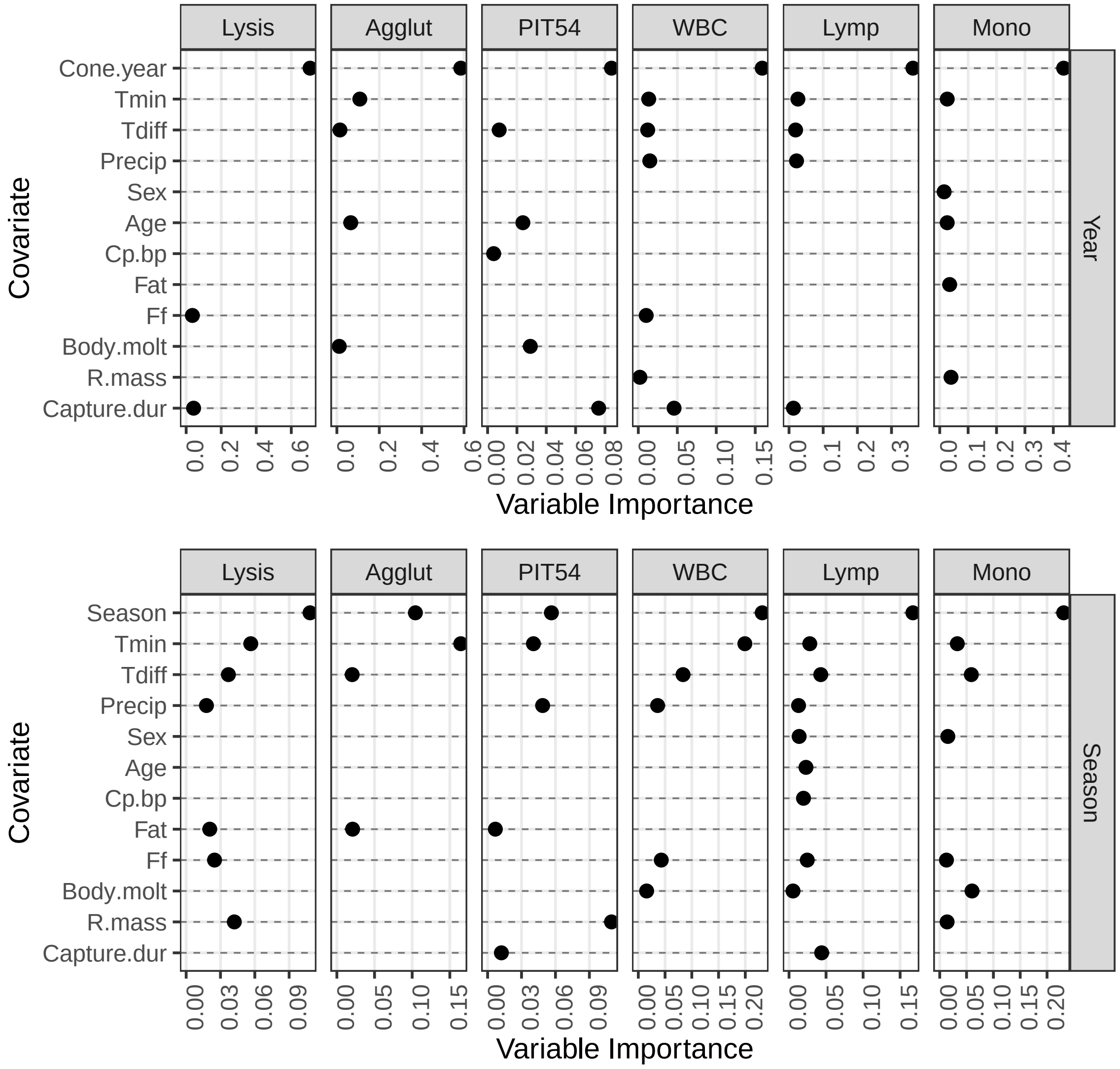

Figure S1: Variable importance for each Random Forest model (RFM, one model per response x time). Covariates with negative variable importance were subsequently removed over 3 serial iterations of model fitting, so that the remaining covariates consistently improve RFM predictive power. To ease comparisons between models, raw importance scores are scaled by model MSE. RFM parameters: mtry=2; ntree=2000.

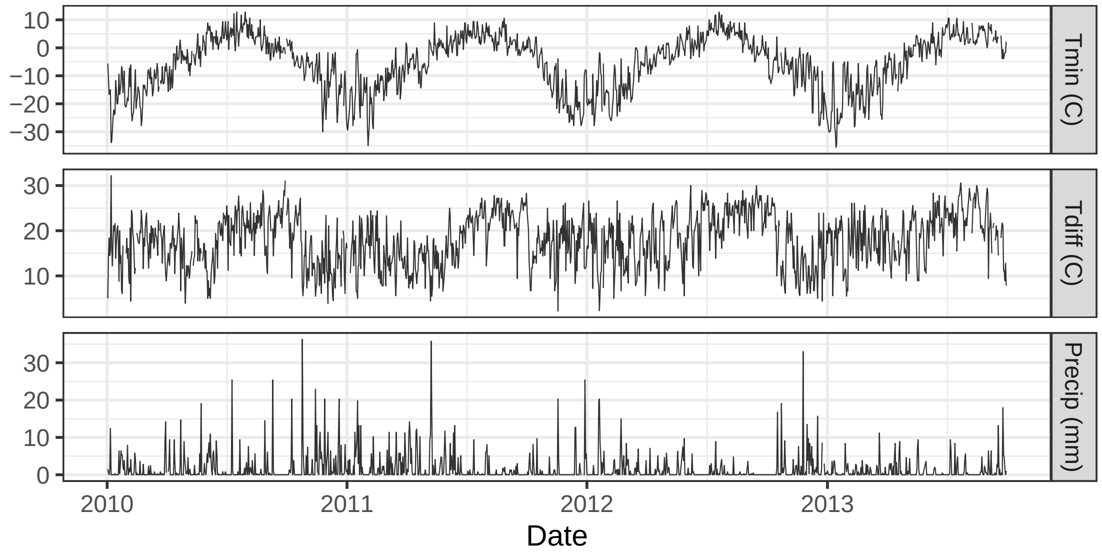

Figure S2: Weather time series from Moose, Wyoming (GHCND Site ID: USC00486428), full period of record. Precipitation shows liquid equivalent.

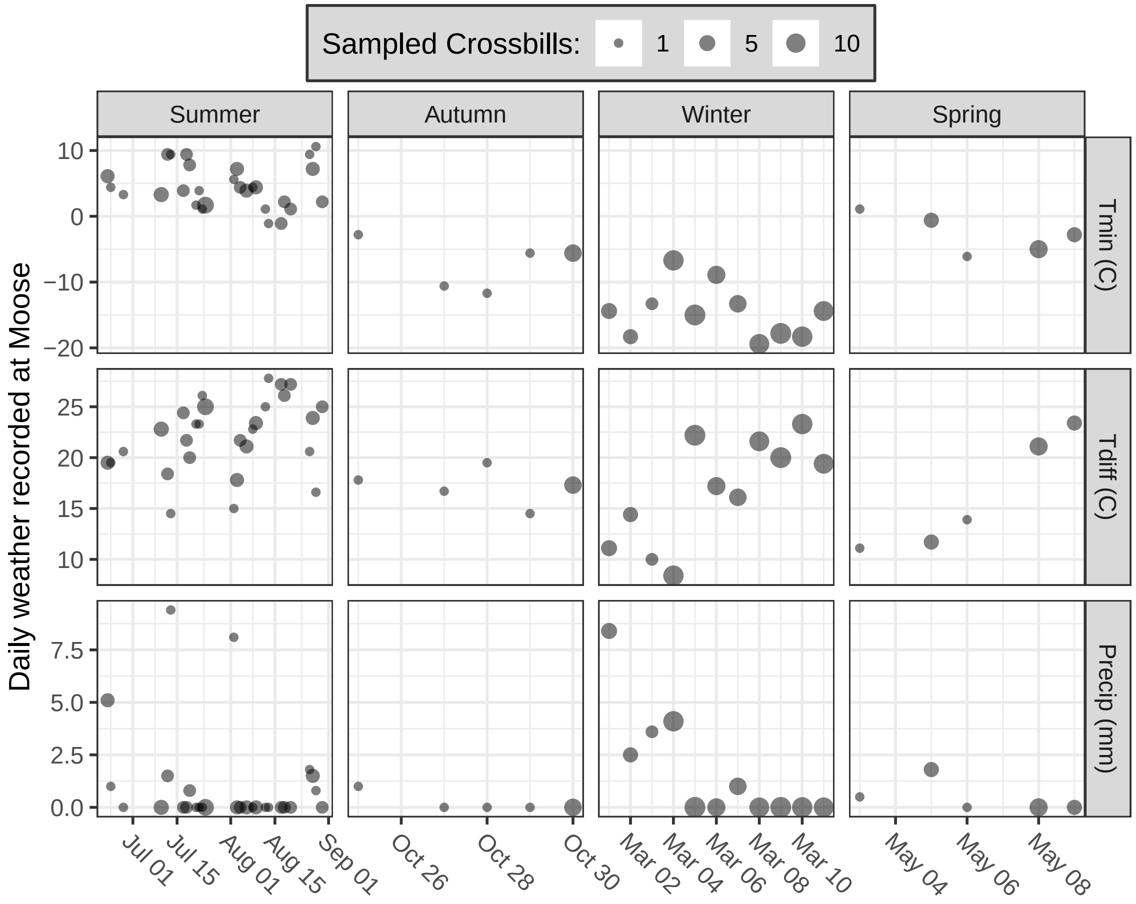

Figure S3: Weather covariates over time, cone year 2011. Point size shows number of captured birds per observation day.

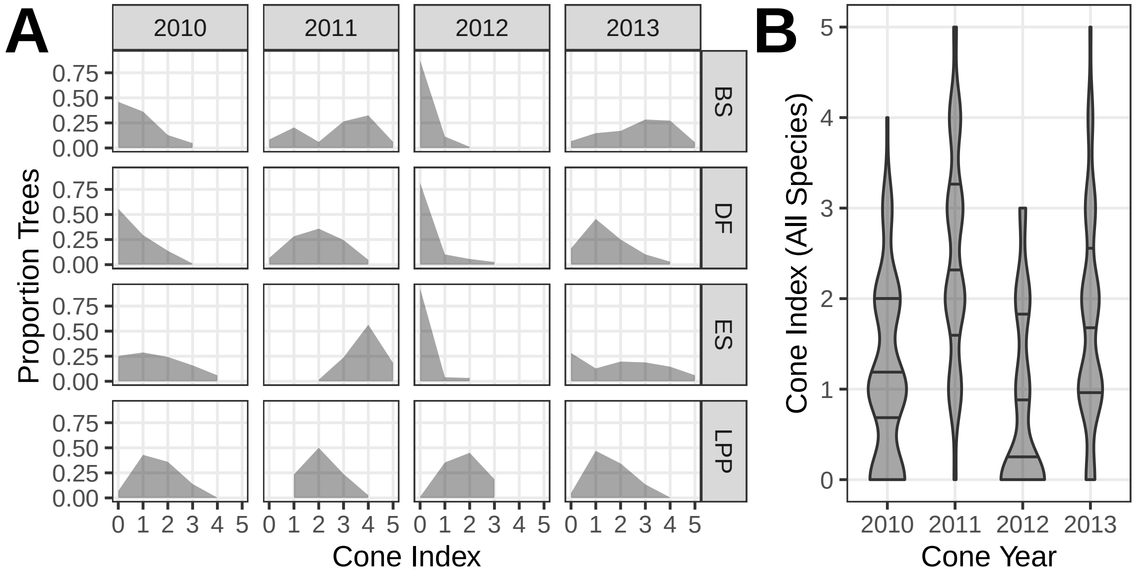

Figure S4: **A.** Distribution of cone score by tree species and cone year. Species include blue spruce (BS, *Picea pungens*), Douglas fir (DF, *Pseudotsuga menziesii*), Engelmann spruce (ES, *Picea engelmannii*), and lodge pole pine (LPP, *Pinus contorta*). **B.** Violin plot of cone index by cone year (Score: 0-5) over all species. Black horizontal lines show the Q1, median, and Q3. Width is proportional to sample count.

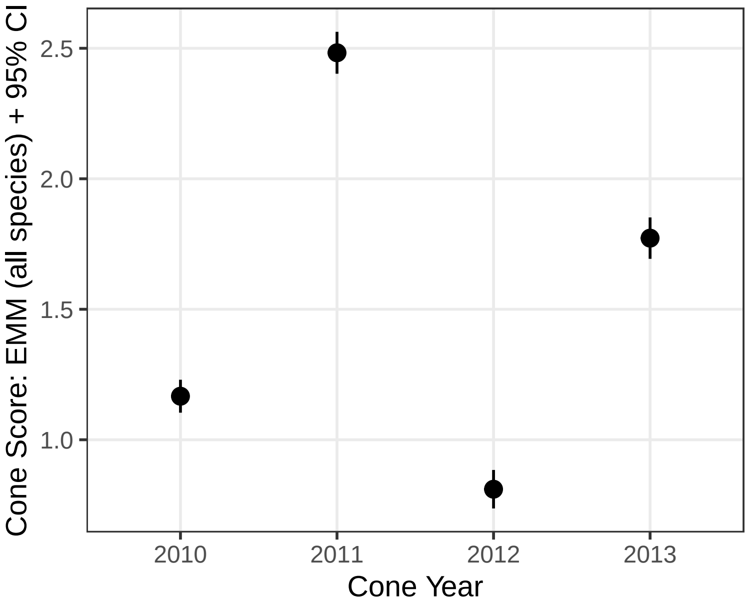

Figure S5: Expected Marginal Means (EMM) of cone score by cone year, showing 95% CI. EMM are computed from an additive linear model of cone score by cone year and tree species (no interactions). All years differ significantly.

```
## `geom_smooth()` using formula 'y ~ x'
```

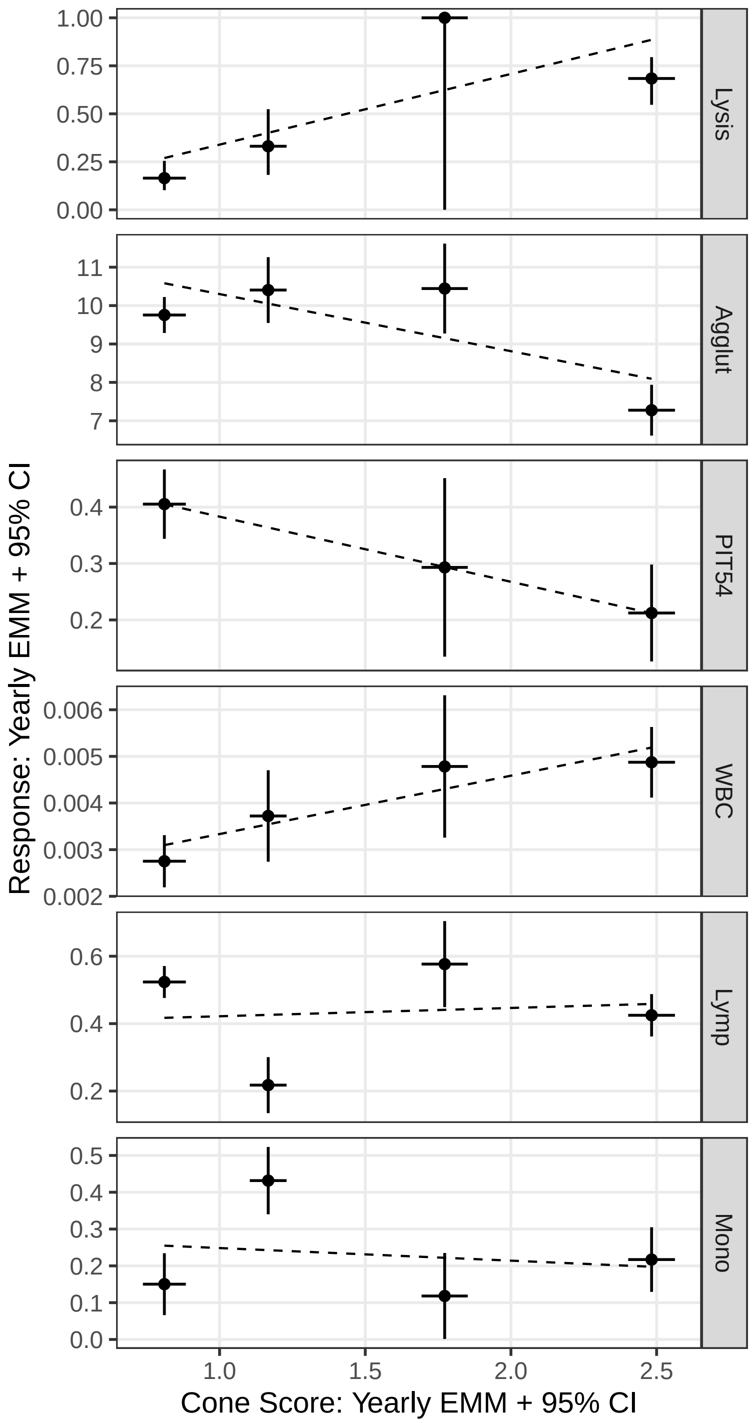

Figure S6: Response yearly EMM versus cone score yearly EMM, for each response (rows). Solid horizontal and vertical lines show respective 95% CI; dashed line shows a best-fit line to respective EMM. See Fig. 2 and Fig. S5 for details.

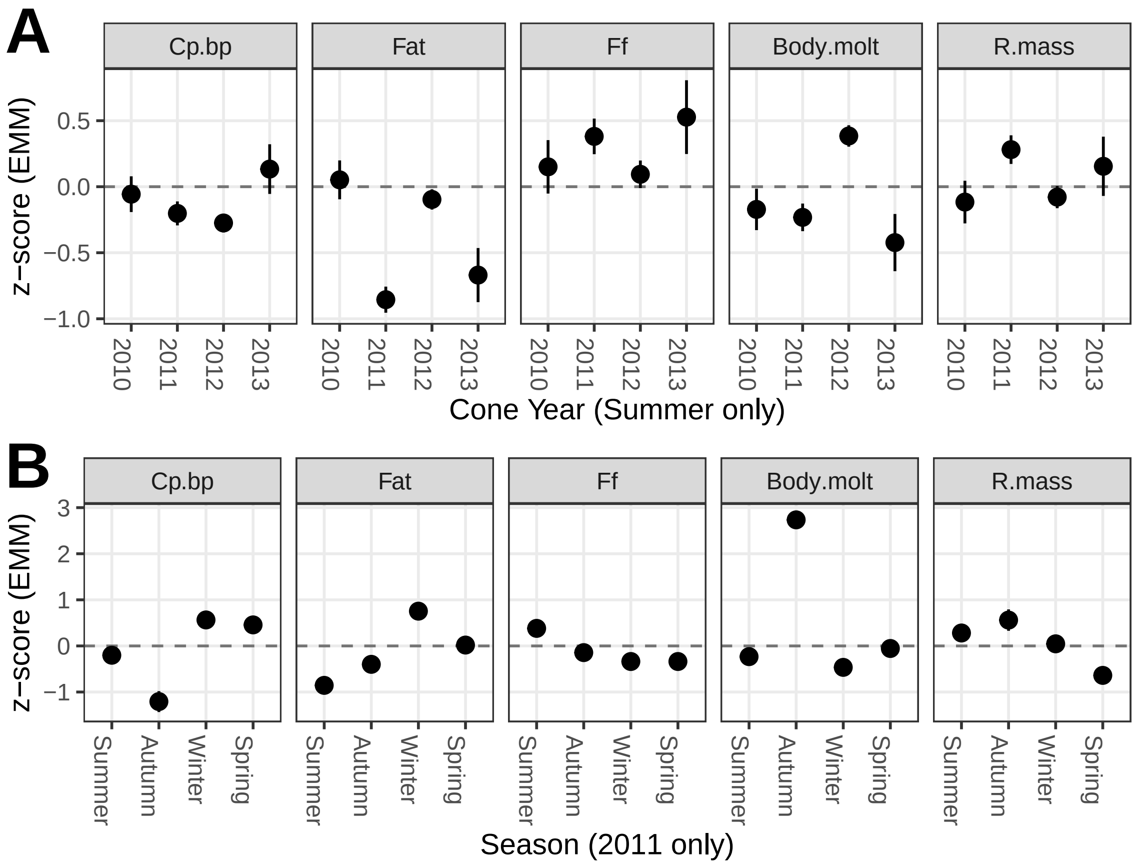

Figure S7: Dependence of continuous physiological covariates on **A**, cone year (Summer observations only) and **B**, season (Cone Year 2011 observations only). Each covariate was z-transformed, and a linear model was constructed using cone year (A) or season (B) as the predictor. The expected marginal mean (EMM) z-score within year (A) and within season (B) are shown, along with 95% CI. Dashed line shows z=0 (covariate sample mean).

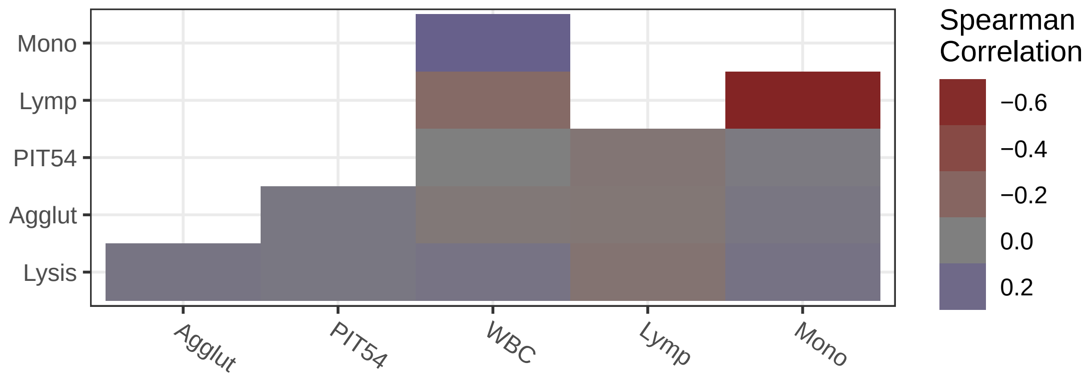

Figure S8: Correlation between measured responses: full period of record.

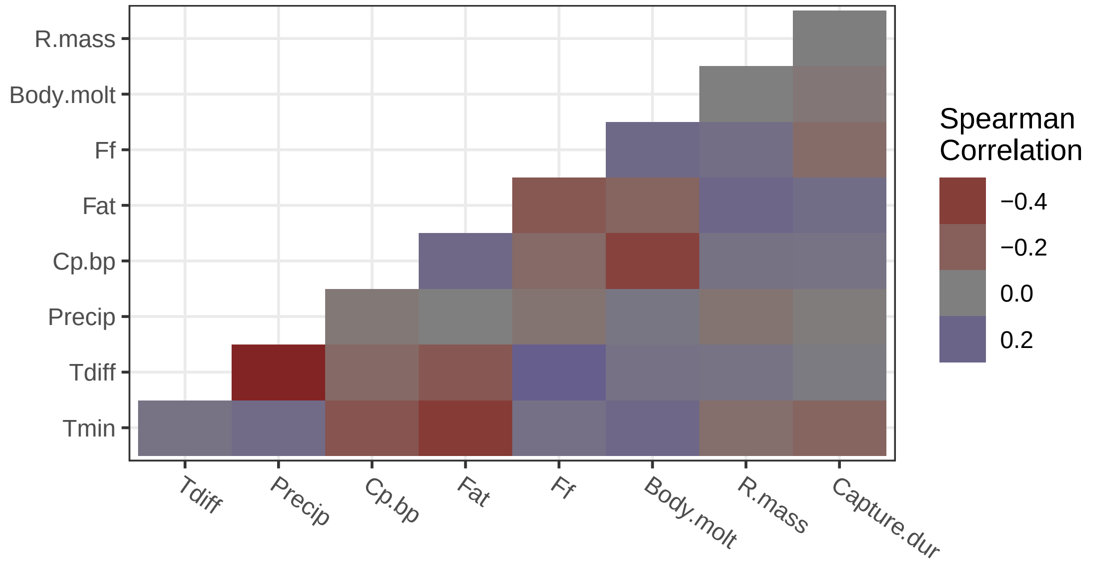

Figure S9: Correlation between covariates: full period of record.
